# Prognostic factors of recovery with medication in patients with taste disorders

**DOI:** 10.1371/journal.pone.0237270

**Published:** 2020-10-01

**Authors:** Yasuyuki Nomura, Teruo Toi, Minoru Ikeda, Atsuo Ikeda, Makoto Tanaka, Takeshi Oshima

**Affiliations:** 1 Department of Otolaryngology-Head and Neck Surgery, Nihon University School of Medicine, Tokyo, Japan; 2 Ikeda ENT Clinic, Tokyo, Japan; University of California, San Francisco, UNITED STATES

## Abstract

**Objectives:**

We aimed to elucidate the prognostic factors of the patients with taste disorders who were treated with popular and common medication in Japan.

**Materials and methods:**

A retrospective study on the medical charts of a total of 255 patients with taste disorders who were treated primarily with oral medication including a zinc agent.

**Results:**

The factors below were significantly linked with poor prognosis: 1) male gender, 2) taste disorders that began 3 months before starting treatment and 3) a severe taste disorder grade at the initial visit.

**Conclusions:**

We have concluded that the prognosis for the patients with taste disorders who were treated by popular and standard medication therapy in Japan recently was significantly linked to gender, the period of 3 months before starting the treatment and the severity of the disorder at the time of diagnosis. In addition, we recognized some limitations we should resolve in further research including a method of measuring “umami” and so on.

**Clinical relevance:**

Better awareness of these factors should be clinically useful when we manage patients with taste disorders. Earlier treatment should be started to cure the symptoms.

## Introduction

The prevalence of taste disorders is high: reported to be 1.1 million in the United States in 1994 [1], and as many as 240,000 in Japan in 2003 [2]. However, in comparison with other sensory organ disorders, taste disorders have not been given proper attention by the medical community. There has hitherto been insufficient research into taste disorders with a limited number of basic or clinical studies. In this research we aimed to estimate the prognostic factor of patients with taste disorders who were treated with popular medication, including an oral zinc agent, in Japan recently.

There are many causes of taste disorders. Among the causes, so far it is said that one of the most significant is the deficiency of trace elements, especially zinc [3–8]. Indeed, certain systemic diseases that precipitate zinc deficiency are accompanied by taste disorders, as observed in studies on patients with diabetes mellitus [9,10], liver cirrhosis [11], renal disorders [12–14], poor growth [15], and Sjögren’s syndrome [16].

Against this background, oral zinc therapy has emerged as a treatment for taste disorders and several clinical studies have been conducted. The types of zinc agent used in these treatment studies include zinc sulfate, zinc gluconate, zinc picolinate, and polaprezinc. The first such report, by Schechter et al. in 1972, was a single-blind study using zinc sulfate in 103 patients and 150 normal subjects [17]. Double-blind studies have been performed using zinc gluconate [18], zinc sulphate [19,20], zinc picolinate [21] and polaprezinc [22–24] in various patient groups. Each of these studies had enrolled patient numbers of approximately 100 (except one study [20], which used only 18 patients) and all reported that zinc has efficacy in alleviating taste disorders.

In recent years, several publications have reported the improvement of taste disorders by oral administration of polaprezinc, a zinc-containing anti-ulcer drug [22–24]. A survey on taste disorders in Japan showed that polaprezinc is the most commonly administered treatment, being used in approximately 70% of hospitals and clinics [2]. The diversity of the role of zinc in the human body and the transmission route of sense has also been studied and recognized [25]. The Medical Guide for Taste Disorder in Japan introduced polaprezinc as one of the oral drugs for taste disorder therapy [26]. In other words, it is said this kind of oral medication therapy, prescriptions including polaprezinc, is a general and popular method for taste disorders in Japan. However, so far there is no report that has researched the prognostic factors of this standard medication therapy. Therefore, in this study, we aimed to investigate the prognostic factors of patients with taste disorders and treat them with the popular medication method.

Clinically, the symptoms of taste disorders are various. Basically, the terms “taste disorder” and “taste disturbance” define the patients who have a decrease in their sense of taste: dysgeusia and hypogeusia, to the complete lack of taste: ageusia. Sometimes, patients complain about the distortion of taste: parageusia, too. In this present study, we use the term “taste disorder” when we include these types of symptoms together.

## Materials and methods

### Subjects

The subjects were 255 patients who visited the Department of Otolaryngology at Nihon University Itabashi Hospital for taste disorders and were treated primarily with an oral zinc agent (polaprezinc). There were 112 males (age range: 21–84 years, mean age: 58.7 ± 15.3 years) and 143 females (age range: 20–82 years, mean age: 56.8 ± 18.1 years). All of the patients were medicated with polaprezinc, but of course some of the patients had been taking some other drugs according to their past histories. Due to this, there were limitations to this study and we needed to take an overviewing stance in order to see the patients’ prognostic factors. This is because we could not perfectly avoid the interference of other drugs. However, it is certain that the results will indicate the prognostic factors of the patients with taste disorders in recent years.

Sometimes patients with taste disorders complain of smell disorders, too. Conversely, patients with smell disorders also sometimes complain of taste disorders. However, we could not examine and include this information about patients with smell disorders in this study.

### Methods

Patients were studied for over 6 months to assess treatment success and were evaluated for various factors that may determine their prognosis. These factors were age, gender, taste disorder etiology, duration of symptoms prior to treatment, the presence or absence of a complaint of a dry mouth, laboratory findings at the initial visit, taste sensitivity in a filter-paper disk (FPD) test at the initial visit, taste disorder severity, and treatment duration.

Taste function was evaluated using an FPD test first described by Tomita et al [27]. A commercially available FPD test kit (Sanwa Kagaku Kogyo, Nagoya, Japan) was used in this study. The subject was asked to describe the taste detected when a circular piece of filter paper, 5 mm in diameter and soaked in a taste solution, was placed on a measurement site on the tongue, such as the bilateral chorda tympani nerve area and the glossopharyngeal nerve area [27]. The tastes used for testing were from the four basic tastes: sweet (sucrose), salty (sodium chloride), sour (tartaric acid) and bitter (quinine), each at one of five different concentrations, as shown in Table 1. The upper limit threshold of normal subjects is set as concentration number 3. The lowest recognizable concentration of the taste solution was obtained by a step-up method, with the different test concentrations acting as thresholds. When the response was absent in the highest test concentration, the threshold was defined as ‘6’ [21]. The taste function in each subject was evaluated by the average value of the threshold measured in the regions of the left and right chorda tympani nerve and the glossopharyngeal nerve.

**Table 1:** Filter-Paper Disk (FPD) test.

Test solution (taste)	Grades and concentration of test solution				
	1	2	3	4	5
Sucrose (sweet)	0.3%	2.5%	10%	20%	80%
NaCl (salty)	0.3%	1.25%	5%	10%	20%
Tartaric acid (sour)	0.02%	0.2%	2%	4%	8%
Quinine hydrochl (bitter)	0.001%	0.02%	0.1%	0.5%	4%

The grade of taste disorder was determined by the average threshold in the FPD test as described by Sakai et al [21] (Table 2). The treatment effect was evaluated as one of two outcomes such as ‘improved’ or ‘not improved’. ‘Improved’ was defined as an improvement of the mean threshold value by 1.0 point or more. All other cases were regarded as showing no curative effect. There is another kind of taste called “umami”, which has become known as a 5th basic taste in recent years [28–31]. However, the clinical examination method to measure “umami” for patients with taste disorders has not been invented, yet. Therefore, we used the FPD with the conventional four kinds of tastes.”

**Table 2:** The grade of taste disorder in the FPD test.

Grade of taste disorder	Taste threshold
Normal	< grade 3.5
Mild	3.5–4.5
Moderate	4.5–5.5
Severe	> 5.5

Polaprezinc is a zinc- and L-carnosine-containing oral medicine manufactured in Japan and widely used for patients with gastric ulcers. This formulation is a white odourless pill containing 75 mg of polaprezinc in one tablet, of which approximately 17 mg is zinc. Normal dosing is to take one pill twice a day, thus delivering 34 mg of zinc per day. The treatment for this study was the normal dosing regimen for oral polaprezinc (150 mg/day, representing 34 mg of zinc). This drug has often been used in the treatment of taste disorders [2] with good efficacy [21, 23]. The patients had continued their coadministered drugs including vitamin agents during the periods.

Statistical analysis was performed using Fischer’s exact probability test and Student’s t-test, for which a p-value of 0.05 or less was considered as significant.

All study methods were approved by the Nihon University Itabashi Hospital Ethics Committee (RK-17041107). All data were fully anonymized and the informed consent of all the patients to use their medical records for this study were approved by the Ethics Committee. There are no conflicts of interest or incentives from any pharmaceutical companies.”

## Results

Table 3 shows the comparison of the various factors between the 166 patients in the group in which treatment was effective and the 89 patients in the group in which the treatment was not effective. In total, the improved cases were 166/255 cases (efficacy ratio: 65.1%) and not improved cases were 89/255 (34.9%). The following factors were significantly different between the two groups: 1) The prognosis was good in females: the ratio of improvement for females was 61.4% and the not improved ratio of males was 53.9%. Both were significantly different and showed better results for females. 2) The prognosis was good in patients who started treatment within 3 months of diagnosis of the taste disorder (improved 43.4%, not improved 30.3%). 3) The results was poor in patients who revealed a severe grade of taste disorder on their first visits’ FPD test: moderate grade patients showed significantly better results (improved 30.7%), but severe grade patients showed significantly poorer results (not improved 57.3%). 4) About the duration of therapy, cases that started treatment within 3 months showed a good prognosis: The cases who responded to our treatment early, indicated a good prognosis (improved 53.0%).

**Table 3:** The breakdown of prognostic factors.

	Improved: 65.1% (166 / 255 cases)	Not improved: 34.9% (89 / 255 cases)	
Gender Male	38.6% (64 / 166 cases)	53.9% (48 / 89 cases)	p < 0.05
Female	61.4% (102 / 166 cases)	46.1% (41 / 89 cases)	p < 0.05
Age	56.8 ± 18.2 (20–84 years old)	56.8 ± 14.3 (25–83 years old)	NS
Duration of taste disorders	7.6 ± 9.1 (1–69 mo.)	10.9 ± 18.6 (1–120 mo.)	NS
Duration: within 3 months	43.4% (72 / 166 cases)	30.3% (27 / 89 cases)	p < 0.05
Dry mouth	28.5% (43 / 151 cases)	30.5% (25 / 82 cases)	NS
**Initial laboratory findings**			
Serum Zn value (μg/dl)	82.4 ± 19.4 (53–190)	84.4 ± 16.9 (60–146)	NS
Cases with low Zn (<70μg/dl)	15.1% (25 / 166 cases)	13.5% (12 / 89 cases)	NS
Cases with low Fe	11.5% (19 / 166 cases)	9.0% (8 / 89 cases)	NS
**FPD test**			
Average FPD test threshold at first visit	5.0 ± 0.9	5.2 ± 1.0	NS
Grades of taste disorders			
Mild	28.3% (47 / 166 cases)	25.8% (23 / 89 cases)	NS
Moderate	30.7% (51 / 166 cases)	16.9% (15 / 89 cases)	p < 0.05
Severe	41.0% (68 / 166 cases)	57.3% (51 / 89 cases)	p < 0.05
**Duration of therapy**			
< 3 months	53.0% (88 / 166 cases)	16.9% (15 / 89 cases)	p < 0.05
4–6 months	24.1% (40 / 166 cases)	24.9% (22 / 89 cases)	NS
7–12 months	17.5% (29 / 166 cases)	46.1% (41 / 89 cases)	NS
> 13 months	5.4% (9 / 166 cases)	12.4% (11 / 89 cases)	NS

Table 4 shows the causes of taste disorder and their efficacy ratio. As Table 3 shows, the total efficacy ratio was 65.1%. We estimated the efficacy ratio of each cause in Table 4. Their ratios were approximately 63–67%. However, C.V.D.: cerebrovascular disease, at 75.1%, and head injury, at 20.0%, were out of this range. Between Tables 3 and 4, there are some difference of the case numbers because of some overlapped cases.

**Table 4:** The causes of taste disorders.

Cause of taste disorders	Number of cases	Improved	Not improved	Improved / Not improved	Efficiency ratio
Idiopathic	86	33.7% (56 / 166 cases)	33.7% (30 / 89 cases)	NS	65.1% (56 / 86 cases)
Drug—induced	86	33.1% (55 / 166 cases)	34.8% (31 / 89 cases)	NS	64.0% (55 / 86 cases)
Zinc deficient (<70μg/dl)	37	15.1% (25 / 166 cases)	13.5% (12 / 89 cases)	NS	67.6% (25 / 37 cases)
Inflamation of U.R.T.	28	11.5% (19 / 166 cases)	10.1% (9 / 89 cases)	NS	67.9% (19 / 28 cases)
Systemic diseases	27	10.2% (17 / 166 cases)	11.2% (10 / 89 cases)	NS	63.0% (17 / 27 cases)
Glossitis	11	4.2% (7 / 166 cases)	4.5% (4 / 89 cases)	NS	63.6% (7 / 11 cases)
C.V.D.	8	3.6% (6 / 166 cases)	2.3% (2 / 89 cases)	NS	75.1% (6 / 8 cases)
Head injury	5	0.6% (1 / 166 cases)	4.5% (4 / 89 cases)	NS	20.0% (1 / 5 cases)

(Total cases are differed from Table 3 because some cases were overlapped. C.V.D: cerebrovascular disease, U.R.T.: upper respiratory tract)

In Table 5, we examined the relation of gender and some factors because there were significant differences in results between males and females as shown in Table 3. The treatment caused improvement in 57.1% of males and 71.3% of females, suggesting that it was significantly more effective in females. The grade of taste disorder was significantly more severe in males, but mean serum zinc values were not significantly different between males and females.

**Table 5:** The gender differences.

	Male 112 cases (43.9%)	Female 143 cases (56.1%)	
Ratio of efficiency	57.1% (64 / 112 cases)	71.3% (102 / 143 cases)	p < 0.05
Duration of taste disorders (months)	9.4 ± 16.6	8.2 ± 9.9	NS
Grade of taste disorders			
Mild	23.2% (26 / 112 cases)	30.1% (43 /143 cases)	NS
Moderate	21.4% (24 / 112 cases)	28.7% (41 / 143 cases)	NS
Severe	55.4% (62 / 112 cases)	41.3% (59 / 143 cases)	p < 0.05
Serum zinc concentration (μg/dl)(mean±SD)	84.3 ± 15.2	82.7 ± 19.9	NS

## Discussion

Polaprezinc is the predominant treatment for taste disorders in Japan and is used in approximately 70% of hospitals and clinics [2]. However, prognostic factors for the popular and standard medication therapy including this zinc agent have not been evaluated. In this study, prognostic factors following administration including this zinc agent were examined.

As shown in Table 3, we confirmed that the factors that would indicate “improved” were 1) female gender, 2) visiting a hospital within 3 months since onset, 3) not showing moderate or severe disturbance on a FPD test and 4) showing signs of recovery within 3 months after starting therapy.

This study included 112 males and 143 females, which was generally consistent with the gender differences previously reported [32]. Regarding gender, this study found that 1) the prognosis was worse for men than for women, and 2) severe cases were seen more frequently in men as shown in Table 5. Taking these two observations together, the reason why the prognosis was poorer in men is unclear. The duration of taste disorder previous to visiting our medical institution and the serum zinc concentration at their first visits, between males and females, were not significantly different. However, the severity of taste disorder at the first visit for males was worse than females. There might be some reason why taste disorder in males tends to have a higher severity than females. It might depend on their background of smoking or some past histories like hypertension, diabetes and so on. Some of their medications for their past histories might chelate the zinc in the way of metabolism. In addition, it is impossible to measure the zinc value contained inside organs. Therefore, there is a possibility the patients’ medication for their past histories influenced the gender difference and it was not shown in the total serum zinc value. However, we could not detect the relations between the patients’ medication for their past histories and the genders. The gender difference in the way of zinc metabolism is also unclear.

Indeed, it has been reported that the prognosis is worse if the start of treatment is delayed after the initial emergence of symptoms [32,33]. Tomita et al. reported that patients who visited after 6 months from the onset of symptoms had poor improvement [33]. In this study, patients who visited within 3 months were significantly more likely to be in the ‘improved’ group, confirming that a longer duration of symptoms before treatment is initiated leads to a worse prognosis.

As for the duration of treatment, 53.0% of the patients in the group for which the treatment was effective showed improvement within 3 months of starting treatment. Therefore, half of the patients for whom zinc therapy was effective can complete treatment in about 3 months, and nearly 80% of patients appeared to be able to complete treatment within 6 months. Therefore, this study suggests that it should be possible to predict at 3 months whether any improvement is being seen. Furthermore, an approximate estimate of the standard treatment period is around 6 months.

As shown in Table 4, there are several causes of taste disorders. In this study, there were no significant differences among the efficiency ratio among these causes. Idiopathic taste disorders show normal laboratory findings, including serum zinc values, and its etiology is still unknown. Zinc deficiency is a situation in which the trigger or cause of the taste disorder is undetermined but manifests itself as reduced serum zinc levels (less than 70 μg/dL). Drug-induced taste disorders occur when another systemic drug causes taste disorders, perhaps owing to zinc chelation [3]. Taste disorders due to glossitis, head injury, cerebrovascular diseases, and upper respiratory inflammation are diagnosed when an alternative trigger or cause cannot be determined. In Table 4, their ratios were approximately 63–67%. There were no significant differences among the causes. However, C.V.D.: cerebrovascular disease, at 75.1%, and head injury, at 20.0%, were out of this range. It seemed that these may be exceptions due to there only being a few patients: 8 cases and 5 cases respectively. Between Tables 3 and 4, there are some differences between the case numbers because of some overlapped cases.

There was no significant difference between the groups of the causes of “idiopathic” and “Zinc deficient” in Table 4. While zinc administration is the logical response to the taste disorders arising owing to zinc deficiency, it may also be effective for idiopathic taste disorder**s**. The reason for this can be inferred as the serum zinc level not reflecting the total amount of zinc in the body, accounting for only 10% to 20% of the zinc in all the blood. Therefore, a state of zinc deficiency in other body tissues should be considered even where serum zinc levels appear normal.

In the same way, in the initial laboratory findings there are no significant differences between the “improved” group and “not improved” group in terms of serum zinc value and cases with low zinc in Table 3. Some of the causes of idiopathic taste disorders are supposed to be latent zinc deficiency. And it has been reported that zinc administration was as effective against this type of taste disorder as it was against that generated by zinc deficiency [18,20]. Therefore, it is clear other factors, such as the severity of taste disorders prior to treatment, have an impact on prognosis. We also suppose that irreversible changes can occur that are not improved by zinc administration, such as abnormalities in zinc metabolism (e.g. malabsorption or increased excretion), although no evidence currently exists to support this speculation.

As for the limitations of this study, we could not avoid the effects of other medication drugs that the patients were originally taking for their past disease histories, as well as habits like smoking, and so on. These factors, including chelation by other medications and various types of zinc metabolism, might have influenced the result of the gender difference. Further research would be desired to resolve this issue.

Limitations also exist with the FPD test. We used a FPD test to examine the degree of taste disorders in this study. The FPD test is a popular method in Japan to measure taste disorders. However, it measures only four basic tastes: sweet, salty, sour and bitter. “Umami” has been recognized as a 5th basic taste in recent years, and its receptors like T1R1, T1R3 and mGluR groups have become known, too [28–31]. However, the method to measure the degree of “umami” clinically has not been invented, yet. This is another limitation of this study, but the invention of such a method to measure “umami” clinically is expected to come in the future.”

Another notable limitation of the FPD test exists, too. The FPD test appears to measure sweet, salty, sour and bitter detection, but not more subtle scents such as "Strawberry" or other distinct scents. Thus, more subtle taste disorders were likely not detected.

Some other limitations include the biases of the samples, the one specific region in Tokyo, Japan they were taken from and the limited size of the samples. It would have been better to have a larger size of samples.”

However, even taking these into consideration, we suppose that the prognostic points above would be valuable information for the clinicians who treat patients with taste disorders.

In addition, the pathological mechanism of taste disorders is not fully understood. For example, the patients undergoing chemoradiation to the head and neck often suffer from taste disorders. The clinical course of their pathology and prognostic factors relating to treatment for their taste disorders is certain to be valuable for further analysis.

## Conclusions

From our results, we have concluded that the prognosis for the patients with taste disorders who were treated by popular and standard medication therapy in Japan recently was significantly linked to gender, the period of 3 months prior to starting the treatment and the severity of the disorder at the time of diagnosis. In addition, further research could resolve some limitations including a method of measuring “umami” and so on. We believe that better awareness of these factors and further research will be clinically useful and important when we manage patients with taste disorders.
